# Theoretical Analysis of Piezoelectric Semiconductor Thick Plates with Periodic Boundary Conditions

**DOI:** 10.3390/mi14122174

**Published:** 2023-11-29

**Authors:** Jueyong Zhu, Mehrdad Negahban, Jie Xu, Rongyu Xia, Zheng Li

**Affiliations:** 1 Department of Mechanics and Engineering Science, College of Engineering, Peking University, Beijing 100871, China; 2 Mechanical and Materials Engineering, University of Nebraska-Lincoln, Lincoln, NE 68588, USA

**Keywords:** piezoelectric semiconductor, multi-layer plates, Stroh formalism, multi-field coupling

## Abstract

Piezoelectric semiconductors, being materials with both piezoelectric and semiconducting properties, are of particular interest for use in multi-functional devices and naturally result in multi-physics analysis. This study provides analytical solutions for thick piezoelectric semiconductor plates with periodic boundary conditions and includes an investigation of electromechanical coupling effects. Using the linearization of the drift-diffusion equations for both electrons and holes for small carrier concentration perturbations, the governing equations are solved by the extended Stroh formalism, which is a method for solving the eigenvalues and eigenvectors of a problem. The solution, obtained in the form of a series expansion with an unknown coefficient, is solved by matching Fourier series expansions of the boundary conditions. The distributions of electromechanical fields and the concentrations of electrons and holes under four-point bending and three-point bending loads are calculated theoretically. The effects of changing the period length and steady-state carrier concentrations are covered in the discussion, which also reflects the extent of coupling in multi-physics interactions. The results provide a theoretical method for understanding and designing with piezoelectric semiconductor materials.

## 1. Introduction

Piezoelectric semiconductors (PSCs) were first discovered in the 1960s and used for designing solid-state electronic devices [1]. With the rapid development of material science and design techniques, PSCs have once again attracted attention for their potential applications in novel mechanical and electrical devices that simultaneously use both the piezoelectric and semiconducting properties. For example, the two new research areas of piezotronics [2,3,4] and piezo-phototronics [5,6,7] are driven and developed based on ZnO micro-/nanowires. In addition, one-dimensional nanostructures of PSCs, such as GaN nanotubes [8] and nanobelts [9], CdS nanowires [10], CdSe nanowires [11], InAs nanowires [12], InN nanorods [13] and others, have been extensively studied and used in the design of nanosensors with special functions [14,15,16].

In addition to one-dimensional nanostructures, there are some studies of two-dimensional PSC materials, such as single-atomic-layer MoS2 and MoSe2 [17,18] and ZnO nanowire thin films [19,20]. It has been observed that two-dimensional materials can achieve better electrical and mechanical properties designable for use as PSC nanostructures and nanosensors [17,21]. Furthermore, two-dimensional nanostructures, like thin films, are more stable, more flexible, and easier to manufacture and have the distinct possibility of combining with other materials to make devices for special functions [22]. Theoretical analysis of two-dimensional PSC structures can provide extensive guidance for discovering new applications of PSCs.

For one-dimensional setups, the multi-field coupling problem resulting from using PSCs can be simplified and solved theoretically. There are studies on the extension and bending problems of single-material fibers and nanowires [23,24,25] and on using two materials [26,27] or more [28,29] with PN junctions to form composite fibers. The theoretical analysis of multi-dimensional PSC problems is more difficult due to more complex equilibrium equations, constitutive relations, and multi-field coupling boundary conditions. In spite of this, there are classic electromechanical coupling problems that have been solved for thin plates [30,31,32], or infinite or semi-infinite domains, such as those for fracture [33,34,35] and wave propagation [36,37,38]. In addition, the studies on the temperature effects of PSCs [39,40,41] and the combinations with other functional materials [42,43] also enrich the understanding of the multi-field coupling effects of structures. However, general solutions for finite-sized geometric shapes with complex boundary conditions can provide much-needed insight for designing PSC devices and sensors [44,45]. In particular, the analysis of two-dimensional PSC plates with finite thickness is suitable for use in designing PSC devices and sensors.

The Stroh formalism provides a practical and elegant way to solve plane problems. The method was proposed by Stroh [46] to solve dislocation, fracture, and steady-state problems of anisotropic elasticity. It has been used by Barnett and Lothe [47] for studying surface waves in piezoelectric crystals and Ting et al. [48] to obtain a complete set of solutions for anisotropic elasticity. Recently, an extended Stroh formalism has been developed for the analysis of piezoelectric materials [49] and used to solve wave propagation problems with periodic boundaries [50,51].

This article extends the Stroh formalism to solve plane problems for PSC materials subject to electromechanical coupling boundary conditions. The extended Stroh formalism is developed for plane problems of PSC materials and a general solution is presented in Section 2. Section 3 demonstrates the solution by looking at two numerical examples of bending and shows the physical field distributions in a thick plate under different boundary conditions. Section 4 studies the solution by considering the influence of the period length, steady-state carrier concentrations, and material parameters. Conclusions are drawn in Section 5.

## 2. A general Solution for a PSC Plate

The following first introduces the basic equations and then the Stroh formalism. Next, interactive boundary conditions are considered and the general PSC solution is developed using Stroh formalism. Finally, the case of constant boundary terms is examined.

### 2.1. The Basic Equations for PSCs

**Figure 1:**
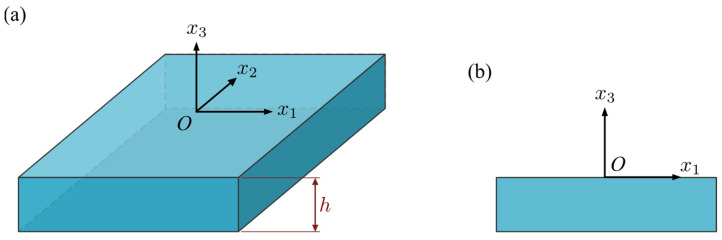
Piezoelectric semiconductor plate: (**a**) 3D view, (**b**) 2D view.

A homogeneous PSC plate in the $Ox_{1}x_{2}$ plane with thickness h is considered here, as shown in Figure 1. The problem is further restricted to a two-dimensional plane-strain state in the $x_{2}$ direction. As a result, the variation of interest is in the $Ox_{1}x_{3}$ plane and defined by boundary conditions given as physical field distributions on the upper and lower surfaces of the plate.

The response of the piezoelectric material is assumed linear and is, thus, described by the constitutive relations:

(1) $$\begin{array}{l} \sigma_{ij}=c_{ijkl}S_{kl}-e_{kij}E_{k},\\ D_{i}=e_{ikl}S_{kl}+\varepsilon_{ik}E_{k}, \end{array}$$

where $\sigma_{ij}$ is the stress tensor, $S_{kl}$ is the strain tensor, $E_{k}$ is the electric field vector, $D_{i}$ is the electric displacement vector, $c_{ijkl}$ is the elastic constant, $e_{kij}$ is the piezoelectric constant, and $\varepsilon_{ik}$ is the dielectric constant [52]. The mechanical strain-displacement relation and the electric field-potential relation are given by:

(2) $$\begin{array}{l} S_{ij}=\frac{1}{2}\left(u_{i,j}+u_{j,i}\right),\\ E_{i}=-\varphi_{,i}, \end{array}$$

where “,” in the subscript denotes a derivative with respect to the spatial coordinate of the noted index.

For semiconductor materials, the current density is an important parameter that includes the influence of two events. One is the drift of the charge carriers caused by existing electric fields and the other is the diffusion caused by the concentration gradient of the carriers. These are captured by the drift-diffusion current relations for holes and electrons given by:

(3) $$\begin{array}{l} J_{i}^{p}=qp\mu_{ij}^{p}E_{j}-qD_{ij}^{p}p_{,j},\\ J_{i}^{n}=qn\mu_{ij}^{n}E_{j}+qD_{ij}^{n}n_{,j}, \end{array}$$

where p and n are, respectively, the concentrations of holes and of electrons; $J_{i}^{p}$ and $J_{i}^{n}$ are the two current densities; $\mu_{ij}^{p}$ and $\mu_{ij}^{n}$ are the associated carrier mobilities; and $D_{ij}^{p}$ and $D_{ij}^{n}$ are the associated carrier diffusion constants, where the superscripts p and n indicate, respectively, the holes and electrons [53]. Here, $q=1.602\times 10^{-19}C$ is the elementary electron charge.

For the static problems of PSC plates, the physical fields are not time-dependent. As such, there are no time-dependent terms in the governing equations, which include the stress equilibrium equation, the electric induction field equation, and the conservation equations of charge for holes and electrons. These are given in the absence of body forces, respectively, as:

(4) $$\begin{array}{l} \sigma_{ij,j}=0,\\ D_{i,i}=q\left(p-n+N_{D}^{+}-N_{A}^{-}\right),\\ J_{i,i}^{p}=0,\\ J_{i,i}^{n}=0, \end{array}$$

where $N_{D}^{+}$ and $N_{A}^{-}$ are, respectively, the concentrations of ionized donors and ionized acceptors [52,53]. If the recombination and generation of electrons and holes are ignored, Equation (4) describes the basic equations governing the static response of piezoelectric semiconductors.

The drift-diffusion current relations given in Equation (3) are not linear, which causes some difficulty in the analysis. To obtain theoretical solutions for semiconductor materials, typically, a first-order perturbation method is used to simplify this equation [23,39]. That is, in Equation (3) each concentration is treated as consisting of a constant term and a small perturbation term, which can be written as:

(5) $$n=n_{0}+\Delta n,\;p=p_{0}+\Delta p,$$

where

(6) $$n_{0}=N_{D}^{+},\;p_{0}=N_{A}^{-}.$$

Assuming that Δp and Δn are much smaller than $p_{0}$ and $n_{0}$, respectively, Equation (3) can be approximated as:

(7) $$\begin{array}{l} J_{i}^{p}=-qp_{0}\mu_{ij}^{p}\varphi_{,j}-qD_{ij}^{p}{\left(\Delta p\right)}_{,j},\\ J_{i}^{n}=-qn_{0}\mu_{ij}^{n}\varphi_{,j}+qD_{ij}^{n}{\left(\Delta n\right)}_{,j}. \end{array}$$

For the following analysis, all the unknown fields will be organized into a single vector that will be termed the generalized displacement vector and denoted by ${\tilde{u}}_{i}$ and all the responses will be organized into a single matrix that will be termed the generalized stress and denoted by ${\tilde{\sigma }}_{ij}$. The specific form of this organization is given as:

(8) $${\tilde{u}}_{i}=\{\begin{array}{ll} u_{i} & i=1,2,3\\ \varphi & i=4\\ \Delta p & i=5\\ \Delta n & i=6 \end{array},\;{\tilde{\sigma }}_{ij}=\{\begin{array}{ll} \sigma_{ij} & i=1,2,3\\ D_{j} & i=4\\ J_{j}^{p} & i=5\\ J_{j}^{n} & i=6 \end{array},\;j=1,2,3.$$

Using this organization, in view of the symmetry of the elastic and piezoelectric tensors and the mechanical strain-displacement and electric field-potential relations of Equation (2), the constitutive relations are given in Equations (1) and (7) can be rewritten as a single generalized constitutive equation. This is given as:

(9) $${\tilde{\sigma }}_{ij}=B_{ijkl}{\tilde{u}}_{k,l},$$

where the coefficients $B_{ijkl}$ are given as:

(10) $$B_{ijkl}=\{\begin{array}{ll} c_{ijkl} & i,k=1,2,3\\ e_{ijl} & i=1,2,3;\;k=4\\ e_{klj} & i=4;\;k=1,2,3\\ -\varepsilon_{jl} & i=k=4\\ -qp_{0}\mu_{jl}^{p} & i=5;\;k=4\\ -qD_{jl}^{p} & i=5;\;k=5\\ -qn_{0}\mu_{jl}^{n} & i=6;\;k=4\\ qD_{jl}^{n} & i=6;\;k=6\\ 0 & \mathrm{others} \end{array},\;j,l=1,2,3.$$

Substituting the generalized constitutive relation, given in Equation (9), into Equation (4), the governing equations for the static response, in terms of the generalized displacement vector, take the form:

(11) $$B_{ijkl}{\tilde{u}}_{k,jl}=q\delta_{i4}\left(\delta_{k5}-\delta_{k6}\right){\tilde{u}}_{k},$$

where $\delta_{ij}$ is the Kronecker delta. This is the form used in the following section to extend the Stroh formalism to the study of the static response of PSC plates.

### 2.2. The Basic Solution by Stroh Formalism

Stroh studied the solution of two-dimensional problems for anisotropic linearly elastic materials [46]. The method proposed by Stroh builds a solution through the analysis of displacement fields that are of fixed direction but have general variation in their magnitude in the plane of the problem. The result is a method that constructs the solution based on the displacement field, as opposed to the Lekhnitskii method [54], which builds the solution based on Airy and Prandtl stress functions. Stroh formalism for solving the problem follows a process similar to its extension provided here.

Stroh formalism is constructed by considering a displacement field that has a fixed direction but is otherwise of a fairly general form. For the problem at hand, we consider a state of plane strain along the $x_{2}$-axis and a general two-dimension displacement in the $Ox_{1}x_{3}$ plane. Stroh formalism suggests constructing a solution to Equation (11) using the generalized displacement field:

(12) $$\tilde{u}=af(z),$$

where a is a constant six-dimensional direction vector, f(z) is a scalar function of the scalar argument $z=x_{1}+px_{3}$, and p is a scalar parameter. This provides the relations:

(13) $$\frac{\partial \tilde{u}}{\partial x_{1}}=a\frac{df(z)}{dz},\;\frac{\partial \tilde{u}}{\partial x_{3}}=pa\frac{df(z)}{dz},$$

and

(14) $$\frac{\partial^{2}\tilde{u}}{\partial x_{1}^{2}}=a\frac{d^{2}f(z)}{dz^{2}},\;\frac{\partial^{2}\tilde{u}}{\partial x_{3}^{2}}=p^{2}a\frac{d^{2}f(z)}{dz^{2}},\;\frac{\partial^{2}\tilde{u}}{\partial x_{1}\partial x_{3}}=pa\frac{d^{2}f(z)}{dz^{2}}.$$

After substitution into Equation (11), one obtains the requirement:

(15) $$\left[Q+p\left(R+R^{\prime }\right)+p^{2}T\right]a\frac{d^{2}f(z)}{dz^{2}}=Aaf(z),$$

where the coefficients are constructed from 6×6 matrices defined as:

(16) $$Q_{ik}=B_{i1k1},\;R_{ik}=B_{i1k3},\;{R^{\prime }}_{ik}=B_{i3k1},\;T_{ik}=B_{i3k3},\;A_{ik}=q\delta_{i4}(\delta_{k5}-\delta_{k6}).$$

The explicit forms of these terms are given as:

$$\begin{array}{l} Q=\left[\begin{array}{cccccc} c_{11} & c_{16} & c_{15} & e_{11} & 0 & 0\\ c_{16} & c_{66} & c_{56} & e_{16} & 0 & 0\\ c_{15} & c_{56} & c_{55} & e_{15} & 0 & 0\\ e_{11} & e_{16} & e_{15} & -\varepsilon_{11} & 0 & 0\\ 0 & 0 & 0 & -qp_{0}\mu_{11}^{p} & -qD_{11}^{p} & 0\\ 0 & 0 & 0 & -qn_{0}\mu_{11}^{n} & 0 & qD_{11}^{n} \end{array}\right],R=\left[\begin{array}{cccccc} c_{15} & c_{14} & c_{13} & e_{31} & 0 & 0\\ c_{56} & c_{46} & c_{36} & e_{36} & 0 & 0\\ c_{55} & c_{45} & c_{35} & e_{35} & 0 & 0\\ e_{15} & e_{14} & e_{13} & -\varepsilon_{13} & 0 & 0\\ 0 & 0 & 0 & -qp_{0}\mu_{13}^{p} & -qD_{13}^{p} & 0\\ 0 & 0 & 0 & -qn_{0}\mu_{13}^{n} & 0 & qD_{13}^{n} \end{array}\right],\\ R^{\prime }=\left[\begin{array}{cccccc} c_{15} & c_{56} & c_{55} & e_{15} & 0 & 0\\ c_{14} & c_{46} & c_{45} & e_{14} & 0 & 0\\ c_{13} & c_{36} & c_{35} & e_{13} & 0 & 0\\ e_{31} & e_{36} & e_{35} & -\varepsilon_{13} & 0 & 0\\ 0 & 0 & 0 & -qp_{0}\mu_{13}^{p} & -qD_{13}^{p} & 0\\ 0 & 0 & 0 & -qn_{0}\mu_{13}^{n} & 0 & qD_{13}^{n} \end{array}\right],T=\left[\begin{array}{cccccc} c_{55} & c_{45} & c_{35} & e_{35} & 0 & 0\\ c_{45} & c_{44} & c_{34} & e_{34} & 0 & 0\\ c_{35} & c_{34} & c_{33} & e_{33} & 0 & 0\\ e_{35} & e_{34} & e_{33} & -\varepsilon_{33} & 0 & 0\\ 0 & 0 & 0 & -qp_{0}\mu_{33}^{p} & -qD_{33}^{p} & 0\\ 0 & 0 & 0 & -qn_{0}\mu_{33}^{n} & 0 & qD_{33}^{n} \end{array}\right],\\ A=\left[\begin{array}{cccccc} 0 & 0 & 0 & 0 & 0 & 0\\ 0 & 0 & 0 & 0 & 0 & 0\\ 0 & 0 & 0 & 0 & 0 & 0\\ 0 & 0 & 0 & 0 & q & -q\\ 0 & 0 & 0 & 0 & 0 & 0\\ 0 & 0 & 0 & 0 & 0 & 0 \end{array}\right]. \end{array}$$

Here, pairs of subscripts associated with the elastic and piezoelectric constants are replaced, as in the Voigt notation, by single subscripts with these rules:11→1, 22→2, 33→3, 23 or 32→4, 13 or 31→5, 12 or 21→6.

In the case of anisotropic elasticity, the right-hand side of Equation (15) is zero; thus, one immediately obtains a solution in the form of an eigenvalue problem. For the extension considered here, a similar solution exists when f(z) takes the form $f(z)=e^{-i\xi z}$ for a constant ξ, termed the characteristic reciprocal length, and the imaginary unit i so that the solution $\tilde{u}=ae^{-i\xi z}$ is formed to satisfy the condition:

(17) $$-\xi^{2}\left[Q+p\left(R+R^{\prime }\right)+p^{2}T\right]a=Aa.$$

This can now be reorganized to obtain the condition:

(18) $$\left[\bar{Q}+p\left(R+R^{\prime }\right)+p^{2}T\right]a=0,$$

for the matrix

(19) $$\bar{Q}=Q+\frac{1}{\xi^{2}}A.$$

The form can now be expressed by an equivalent eigen equation, known as the extended Stroh’s eigenvalue problem, given by:

(20) $$N\left[\begin{array}{c} a\\ b \end{array}\right]=p\left[\begin{array}{c} a\\ b \end{array}\right],$$

for the unknown vector ${\left[\begin{array}{cc} a & b \end{array}\right]}^{T}$, where the 12×12 matrix N and the six-dimensional vector b are:

(21) $$N=\left[\begin{array}{cc} -T^{-1}R^{\prime } & T^{-1}\\ -\bar{Q}+RT^{-1}R^{\prime } & -RT^{-1} \end{array}\right],\;b=\left[R^{\prime }+pT\right]a.$$

If this eigenvalue problem is non-degenerate, there will be six complex conjugate pairs that identify twelve linearly independent eigenvectors. Solving Equation (20) for a given ξ provides a base for constructing a general solution by a linear combination based on the Stroh formalism. Letting β denote an index from 1 to 6, each pair of eigenvalues will be denoted by $\left(p_{\beta },{\bar{p}}_{\beta }\right)$ and their associated eigenvectors by $\left(a_{\beta },{\bar{a}}_{\beta }\right)$. The solution then takes the form:

(22) $$\tilde{u}=\sum_{\beta =1}^{6}c_{\beta }a_{\beta }e^{-i\xi \left(x_{1}+p_{\beta }x_{3}\right)}+\sum_{\beta =1}^{6}d_{\beta }{\bar{a}}_{\beta }e^{-i\xi \left(x_{1}+{\bar{p}}_{\beta }x_{3}\right)},$$

where $c_{\beta }$ and $d_{\beta }$ are undetermined coefficients. Using this solution in the generalized constitutive relation given in Equation (9) allows one to evaluate the generalized traction on a surface that is normal along $x_{3}$ through the relation:

(23) $$\tilde{t}={\tilde{\sigma }}_{i3}=-i\xi \left(\sum_{\beta =1}^{6}c_{\beta }b_{\beta }e^{-i\xi \left(x_{1}+p_{\beta }x_{3}\right)}+\sum_{\beta =1}^{6}d_{\beta }{\bar{b}}_{\beta }e^{-i\xi \left(x_{1}+{\bar{p}}_{\beta }x_{3}\right)}\right),$$

which is the same as the displacement in a linear combination using $b_{\beta }$. The solution to the problem requires one to obtain the undetermined coefficients by using specific boundary conditions.

### 2.3. Boundary Conditions

The generalized unknowns may be subject to the Dirichlet, Neumann, or a linear mix of these conditions. Physically, these correspond, respectively, with a generalized displacement, a generalized traction, or a linear combination. For a homogeneous PSC plate with thickness h, as shown in Figure 1, the boundary conditions on the upper and lower surfaces (i.e., $x_{3}=0,-h$) can be written in a unified notation as:

(24) $$\begin{array}{l} I_{u}^{1}\tilde{u}\left(x_{1},0\right)+I_{t}^{1}\tilde{t}\left(x_{1},0\right)=F^{1}\left(x_{1}\right),\\ I_{u}^{2}\tilde{u}\left(x_{1},-h\right)+I_{t}^{2}\tilde{t}\left(x_{1},-h\right)=F^{2}\left(x_{1}\right), \end{array}$$

where $F^{1}(x_{1})$ and $F^{2}(x_{1})$ are six-dimensional vector-valued functions of $x_{1}$ and $I_{u}^{1},\;I_{t}^{1},\;I_{u}^{2}$, and $I_{t}^{2}$ are 6×6 diagonal matrices that satisfy $I_{u}^{1}+I_{t}^{1}=I_{u}^{2}+I_{t}^{2}=I_{6}$, where $I_{6}$ is the six-by-six identity matrix. In this unified form, the condition $I_{u}^{1}=I_{6}$ and $I_{t}^{1}=0$ represents a generalized displacement boundary condition while the $I_{u}^{1}=0$ and $I_{t}^{1}=I_{6}$ condition represents a generalized stress boundary condition. Here we only considered the cases where the elements of these diagonal matrices were 0 or 1. The more general case can be found in [48].

**Figure 2:**
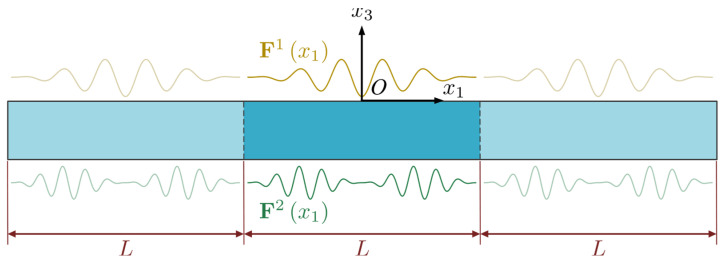
Piezoelectric semiconductor plate with periodical boundary conditions.

Finite PSC plates are commonly utilized in practice for smart devices or sensors that are subject to periodic conditions. In this case, the functions $F^{1}(x_{1})$ and $F^{2}(x_{1})$ can describe the periodical distributions of physical fields on the upper and lower surfaces of the PSC plate, as shown in Figure 2. For the period length L along the $x_{1}$ direction, this transforms the infinite PSC plate problem into a finite one and makes the components of $F^{1}(x_{1})$ and $F^{2}(x_{1})$ into periodic functions of period L.

### 2.4. The General Solution

The infinite PSC plate with periodical boundary conditions on both surfaces can be solved using Batra’s method for imposing boundary conditions using the Fourier series decomposition method [55,56]. In this case, the periodic functions $F^{1}(x_{1})$ and $F^{2}(x_{1})$ are described by their Fourier series, given by:

(25) F1(x1)=A012+Re∑α=1∞(Aα1+iBα1)e−iαkx1,F2(x1)=A022+Re∑α=1∞(Aα2+iBα2)e−iαkx1,

where $A_{\alpha }^{1}$, $B_{\alpha }^{1}$, $A_{\alpha }^{2}$, and $B_{\alpha }^{2}$ are vectors of trigonometric Fourier coefficients. For the convenience of the subsequent solution, it is written in a complex exponential form that retains only the real part due to the physical meaning. The Fourier series expansion separates the solution into two parts, one being constant and the other having exponential terms. These parts are solved as follows.

#### 2.4.1. The Solution for a Constant Loading Term

When the physical boundary conditions are constants, the problem reduces to the simpler one-dimensional case. That is, the physical fields become functions of only $x_{3}$. In this case, the governing equation, Equation (11), transforms into an equation for displacement vector $u_{0}(x_{3})$, given by:

(26) $$T{u^{\prime \prime }}_{0}=Au_{0}.$$

This is a linear second-order ordinary differential equation. It is similar to the characteristic equation for steady vibration for a multi-degree-of-freedom system with only one non-zero eigenvalue. It has a general solution of the form:

(27) $${\tilde{u}}_{0}=C_{0}+C_{1}x_{3}+a_{0}\left[c_{6}e^{k_{0}x_{3}}+c_{12}e^{-k_{0}(x_{3}+h)}\right],$$

with the traction

(28) $${\tilde{t}}_{0}=TC_{1}+k_{0}b_{0}\left[c_{6}e^{k_{0}x_{3}}-c_{12}e^{-k_{0}(x_{3}+h)}\right],$$

where

(29) $$\begin{array}{l} C_{0}={\left[c_{1},c_{2},c_{3},c_{4},c_{5},c_{5}\right]}^{T},\\ C_{1}={\left[c_{7},c_{8},c_{9},c_{10},c_{11},c_{11}\right]}^{T},\\ k_{0}=\sqrt{q\left(\frac{p_{0}\mu_{33}^{p}}{D_{33}^{p}}+\frac{n_{0}\mu_{33}^{n}}{D_{33}^{n}}\right)\frac{\left|T_{3}\right|}{-\left|T_{4}\right|}},\;\\ a_{0}={\left[-{\left(T_{3}^{-1}\right)}_{1j}T_{j4},-{\left(T_{3}^{-1}\right)}_{2j}T_{j4},-{\left(T_{3}^{-1}\right)}_{3j}T_{j4},1,-\frac{p_{0}\mu_{33}^{p}}{D_{33}^{p}},\frac{n_{0}\mu_{33}^{n}}{D_{33}^{n}}\right]}^{T},\\ b_{0}={\left[0,0,0,\frac{\left|T_{4}\right|}{\left|T_{3}\right|},0,0\right]}^{T}. \end{array}$$

Here, $c_{1}$ to $c_{12}$ are undetermined coefficients, $T_{3}$ and $T_{4}$ are the third and fourth principal submatrix of T, $k_{0}$ is the non-zero eigenvalue, and $a_{0}$ is the corresponding eigenvector. With this consideration, Equation (24) takes the form:

(30) $$\begin{array}{l} I_{u}^{1}C_{0}+I_{t}^{1}TC_{1}+\left(I_{u}^{1}a_{0}+k_{0}I_{t}^{1}b_{0}\right)c_{6}+(I_{u}^{1}a_{0}-k_{0}I_{t}^{1}b_{0})e^{-k_{0}h\;}c_{12}=\frac{A_{0}^{1}}{2},\\ I_{u}^{2}C_{0}-I_{u}^{2}C_{1}h+I_{t}^{2}TC_{1}+\left(I_{u}^{2}a_{0}+k_{0}I_{t}^{2}b_{0}\right)e^{-k_{0}h\;}c_{6}+(I_{u}^{2}a_{0}-k_{0}I_{t}^{2}b_{0})c_{12}=\frac{A_{0}^{2}}{2}. \end{array}$$

These represent twelve equations to be solved for twelve unknown parameters. The problem is solvable when both the upper and lower surfaces are defined by generalized displacement boundary conditions or when one is replaced by a surface with stress boundary conditions. As expected, if both surfaces are described by applying generalized stress conditions, there will be an undetermined translation due to the premise of the equilibrium of the physical fields. For example, tractions on both surfaces should be equal due to the force balance. In this situation, zero generalized displacements can be set on the midpoint ($x_{3}=-h/2$) and used as a supplementary condition [23,26]; this can be written as:

(31) $$I_{t}^{1}I_{t}^{2}\left[C_{0}-\frac{h}{2}C_{1}+a_{0}e^{-\frac{k_{0}h}{2}\;}\left(c_{6}+c_{12}\right)\right]=0.$$

This allows for the solving of all twelve undetermined coefficients, irrespective of the kind of boundary conditions provided.

#### 2.4.2. The Solution for the Exponential Loading Terms

The exponential terms in the boundary conditions given in Equation (25) can be written for the αth term from the basic solution based on the Stroh formalism given in Equation (22). This provides that the displacement ${\tilde{u}}_{\alpha }$ is the Stroh formalism solution for ξ=αk as:

(32) $${\tilde{u}}_{\alpha }=\overset{6}{\underset{\beta =1}{\sum }}\left[c_{\alpha \beta }a_{\alpha \beta }e^{-i\alpha k(x_{1}+p_{\alpha \beta }x_{3})}+d_{\alpha \beta }{\bar{a}}_{\alpha \beta }e^{-i\alpha k(x_{1}+{\bar{p}}_{\alpha \beta }x_{3}+{\bar{p}}_{\alpha \beta }h)}\right],$$

and the associated traction

(33) $${\tilde{t}}_{\alpha }=\overset{6}{\underset{\beta =1}{\sum }}\left(-i\alpha k\right)\left[c_{\alpha \beta }b_{\alpha \beta }e^{-i\alpha k(x_{1}+p_{\alpha \beta }x_{3})}+d_{\alpha \beta }{\bar{b}}_{\alpha \beta }e^{-i\alpha k(x_{1}+{\bar{p}}_{\alpha \beta }x_{3}+{\bar{p}}_{\alpha \beta }h)}\right].$$

Substituting these into Equation (24) provides the twelve relations for calculating the undetermined coefficients $c_{\alpha \beta }$ and $d_{\alpha \beta }$. These relations are:

(34) $$\begin{array}{l} \overset{6}{\underset{\beta =1}{\sum }}\left(I_{u}^{1}a_{\alpha \beta }-i\alpha kI_{t}^{1}b_{\alpha \beta }\right)c_{\alpha \beta }+\overset{6}{\underset{\beta =1}{\sum }}\left(I_{u}^{1}{\bar{a}}_{\alpha \beta }+i\alpha kI_{t}^{1}b_{\alpha \beta }\right)e^{-i\alpha k{\bar{p}}_{\alpha \beta }h}d_{\alpha \beta }=A_{\alpha }^{1}+iB_{\alpha }^{1},\\ \overset{6}{\underset{\beta =1}{\sum }}\left(I_{u}^{2}a_{\alpha \beta }-i\alpha kI_{t}^{2}b_{\alpha \beta }\right)e^{i\alpha kp_{\alpha \beta }h}c_{\alpha \beta }+\overset{6}{\underset{\beta =1}{\sum }}\left(I_{u}^{2}{\bar{a}}_{\alpha \beta }+i\alpha kI_{t}^{2}b_{\alpha \beta }\right)d_{\alpha \beta }=A_{\alpha }^{2}+iB_{\alpha }^{2}. \end{array}$$

#### 2.4.3. The General Solution

Combining the results from Equations (27) and (32) and using the Fourier series expansion provides a general periodic solution given the constants calculated from Equations (30) and (34). This results in a generalized periodic displacement and the $x_{3}$-surface traction in the form:

(35) u˜=C0+C1x3+a0[c6ek0x3+c12e−k0(x3+h)] +Re∑α=1∞∑β=16[cαβaαβe−iαk(x1+pαβx3)+dαβa¯αβe−iαk(x1+p¯αβx3+p¯αβh)],t˜=TC1+k0b0[c6ek0x3−c12e−k0(x3+h)] +Re∑α=1∞∑β=16(−iαk)[cαβbαβe−iαk(x1+pαβx3)+dαβb¯αβe−iαk(x1+p¯αβx3+p¯αβh)].

### 2.5. Degeneration from PSCs to Piezoelectric and Elastic Solutions

The analysis method proposed can be used to solve similar problems for degenerate forms of PSC materials, such as piezoelectric semiconductors (p- or n-type) and simple piezoelectric and elastic materials. In these degenerations, the dimensions of the generalized displacement vector and generalized stress tensor in Equation (8) reduce to 5, 4, and 3, accordingly. For example, the general solution for a thick piezoelectric plate is given by:

(36) u˜piezo=C0*+C1*x3+Re∑α=1∞∑β=14[cαβ*aβ*e−iαk(x1+pβ*x3)+dαβ*a¯β*e−iαk(x1+p¯β*x3+p¯β*h)],t˜piezo=T*C1*+Re∑α=1∞∑β=14(−iαk)[cαβ*bβ*e−iαk(x1+pβ*x3)+dαβ*b¯β*e−iαk(x1+p¯β*x3+p¯β*h)].

Here, $C_{0}^{*}$ and $C_{1}^{*}$ are undetermined four-dimensional vectors and ${\left[\begin{array}{cc} a_{\beta }^{*} & b_{\beta }^{*} \end{array}\right]}^{T}$ and $p_{\beta }$ are the eigenvectors and eigenvalues of Stroh’s problem for the piezoelectric material, which has been proposed in refs. [49,57].

## 3. Numerical Example for a PSC Plate under Four-Point Bending

The following demonstrates the proposed solution method applied to a transversely isotropic zinc oxide (ZnO) plate under four-point bending. ZnO is a widely studied PSC material, with its properties described in Table 1, as given in [58]. The axis of transverse isotropy of ZnO is assumed to be aligned with the $x_{3}$-axis of the plate so that the planes of isotropy are horizontal while the axis of transverse isotropy is vertical. The drift and diffusion of holes and electrons are assumed isotropic (i.e., $\mu_{11}^{p,n}=\mu_{33}^{p,n}$, $\mu_{13}^{p,n}=0$; $D_{11}^{p,n}=D_{33}^{p,n}$, $D_{13}^{p,n}=0$). The carrier mobilities $\mu_{33}^{p,n}$ and carrier diffusion constants $D_{33}^{p,n}$ are selected to satisfy the Einstein relation:

(37) $$\frac{\mu_{ij}^{p}}{D_{ij}^{p}}=\frac{\mu_{ij}^{n}}{D_{ij}^{n}}=\frac{q}{k_{B}T}=38.46\;V^{-1},$$

where $k_{B}$ is the Boltzmann constant and T is the absolute temperature, here taken to be 300 K. This ratio will be used instead of carrier mobilities and carrier diffusion constants during the calculation.

Our purpose is to study the physical field distributions of a PSC plate under bending conditions. We consider a PSC plate of nanoscale with the dimensions h=0.05 μm and L=1.2566 μm and with applied local traction distributions on the upper and lower surface. A four-point bending-like load is applied to the positive half-segment of the plate and the inverse is applied to the negative half-segment. Due to the symmetry, only the response on the positive half-segment is presented here.

**Figure 3:**
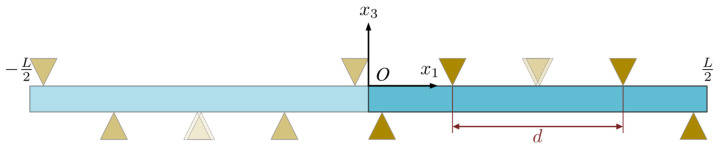
Schematic diagram of four-point bending.

For the demonstration, we consider the mechanical four-point loading setup, as is schematically shown in the diagram given in Figure 3, which is electrically isolated and with no current flow. This mechanical loading induces a bending response that is characterized by the partition of the beam into three segments that include two transition shoulders and one constant-moment central segment. The two transition shoulders are constant shear load segments that result in linearly increasing moments, which start from zero and increase toward the center. The central segment is a zero-shear load segment that results in a constant (pure) moment. As such, the demonstration simultaneously shows the multi-physics interactions for segments at a constant shear load and constant bending moment.

To capture mechanical four-point bending in the analysis, the point loads are modeled as triangular distributed loads of height $\sigma_{0}$ and width h. As shown in Figure 3, the loads on the lower surface are fixed and centered around points $x_{1}=h/2$ and $x_{1}=\left(L-h\right)/2$ while those on the upper surface will be varied by selecting the loading distance d while keeping the loading symmetrical about the midpoint $x_{1}=L/2$. This loading method preserves continuity in the traction load on each of the two surfaces and preserves the centrosymmetric loading of the positive and negative half-axis. In the demonstration, the peak distributed stress is taken as $\sigma_{0}=1\times 10^{6}{\;N/m}^{2}$. Using the function H(x), this is defined as:

(38) $$H(x)=\{\begin{array}{l} 1-\frac{2\left|x\right|}{h},\;x\in \left[-\frac{h}{2},\frac{h}{2}\right],\\ 0,\;\mathrm{else}. \end{array}$$

By this take, d=L/4, which means the loads on the upper surface are centered around points $x_{1}=L/8$ and $x_{1}=3L/8$. The specific four-point bending PSC plate boundary conditions used are:

(39) $$\begin{array}{l} t_{3}(x_{1},-h)=-\sigma_{0}\left[H\left(x_{1}+\frac{3L}{8}\right)+H\left(x_{1}+\frac{L}{8}\right)+H\left(x_{1}-\frac{h}{2}\right)+H\left(x_{1}-\frac{L-h}{2}\right)\right],\\ t_{3}(x_{1},0)=-\sigma_{0}\left[H\left(x_{1}+\frac{L-h}{2}\right)+H\left(x_{1}+\frac{h}{2}\right)+H\left(x_{1}-\frac{L}{8}\right)+H\left(x_{1}-\frac{3L}{8}\right)\right],\\ D_{3}(x_{1},-h)=D_{3}(x_{1},0)=0,\\ J_{3}^{p}(x_{1},-h)=J_{3}^{p}(x_{1},0)=0,\\ J_{3}^{n}(x_{1},-h)=J_{3}^{n}(x_{1},0)=0, \end{array}$$

with the initial concentrations set as $p_{0}=n_{0}=5\times 10^{21}{\;m}^{-3}$. The Fourier series expansion of the boundary conditions, given in Equation (25), were truncated at appropriate α to ensure the results have sufficient accuracy and that the solution converges. Here, the distribution of the electric displacement component $D_{3}$ is chosen as the object of the convergence study. We incremented α from 0 to 150 using increments of 10. The relative error $\varepsilon_{D}$ was generated by comparing the results of two consecutive calculations, as defined by:

$$\varepsilon_{D}=\frac{\max\left|D_{3}^{\left(\alpha \right)}-D_{3}^{\left(\alpha -10\right)}\right|}{\max D_{3}^{\left(\alpha \right)}-\min D_{3}^{\left(\alpha \right)}}.$$

**Figure 4:**
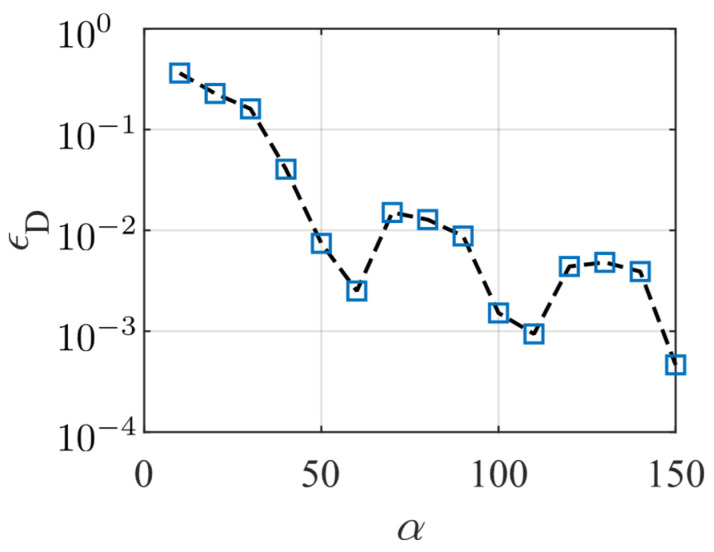
The relationship between the relative error $\varepsilon_{D}$ and α.

Figure 4 shows the relationship between the relative error $\varepsilon_{D}$ and α. The results show consistent convergence when α is greater than 50. The value of $\varepsilon_{D}$ is less than 1% when α is greater than 100. In subsequent calculations, we take α=100.

**Figure 5:**
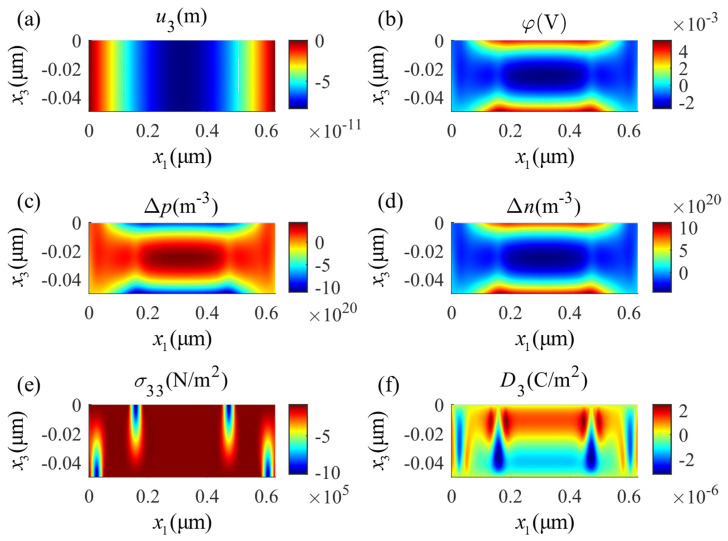
Distributions of physical fields in the four-point bending state: (**a**) mechanical displacement $u_{3}$, (**b**) electric potential φ, (**c**) perturbation of hole concentrations Δp, (**d**) perturbation of electron concentrations Δn, (**e**) stress component $\sigma_{33}$, (**f**) electric displacement $D_{3}$.

The distribution of the physical fields in the positive half-axis is shown in Figure 5. The zero points of displacement and potential are located at the center of the lower surface. Figure 5a provides the displacement component $u_{3}$ and Figure 5e shows the distribution of the stress component $\sigma_{33}$, both working along the thickness direction. As shown in Figure 5b, the electromechanical coupling of the material makes the potential distribution decay from the outside to inside of the plate. This change shows a sharp transition near the top and bottom boundaries and tends to be uniform inside the beam. The electric displacement is shown in Figure 5f and indicates extreme values that change sharply around the mechanical loading points. The perturbation of electron concentration Δn, shown in Figure 5d, indicates a similar distribution to the electric potential; meanwhile, the perturbation of hole concentration Δp, indicated in Figure 5c, shows similar but opposite values. This follows from the fact that there is no outward/inward current density on the surface and that the drift and diffusion of the particles are uniform. Hence, the gradient of the concentrations of the holes and electrons will be consistent with the electric field. In summary, the mechanical load creates a complex electric field and carrier concentration distribution in the PSC plate, which is the result of the multi-physics interactions.

We next consider the special circumstance of d=0 so that the two center loads merge. This creates three-point bending and results in maximally expanding the two shoulder segments and eliminating the central constant moment segment. As a result, the three-point bending condition better exposes the multi-physics interaction when the beam is under a constant shear load and a linearly varying bending moment that is at its maximum at the center of the beam. For three-point bending, the boundary stress distributions in Equation (39) are replaced with:

(40) $$\begin{array}{l} t_{3}(x_{1},-h)=-\sigma_{0}\left[2H\left(x_{1}+\frac{L}{2}\right)+H\left(x_{1}-\frac{h}{2}\right)+H\left(x_{1}-\frac{L-h}{2}\right)\right],\\ t_{3}(x_{1},0)=-\sigma_{0}\left[H\left(x_{1}+\frac{L-h}{2}\right)+H\left(x_{1}+\frac{h}{2}\right)+2H\left(x_{1}-\frac{L}{2}\right)\right]. \end{array}$$

**Figure 6:**
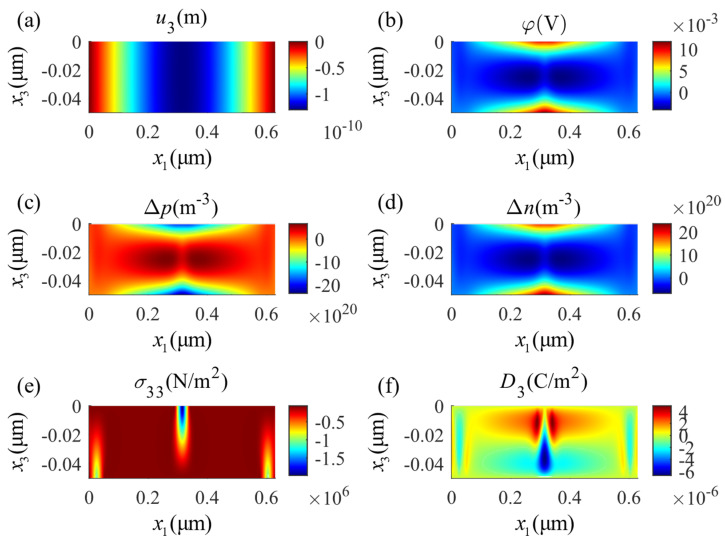
Distributions of physical fields in the three-point bending state: (**a**) mechanical displacement $u_{3}$, (**b**) electric potential φ, (**c**) perturbation of hole concentrations Δp, (**d**) perturbation of electron concentrations Δn, (**e**) stress component $\sigma_{33}$, (**f**) electric displacement $D_{3}$.

The distributions of the same physical fields described for four-point bending in Figure 5 are shown for three-point bending in Figure 6. Comparing these two figures, one can see that local loading applied on the surface diffusely changes the potential and the carrier concentration distributions. Physically, the piezoelectricity and the conductivity of the PSC material are related to the carrier concentration; however, the carrier distribution is related to the potential distribution. This reflects the complex multi-field coupling effects in the PSC material. Comparing these two examples, the minimum value of the potential in the four-point bending appears at the center of the constant moment segment while it appears in the three-point bending, due to the disappearance of the constant moment segment, at the two positions with the minimum value of the potential that are symmetrically located close to the center.

## 4. The Discussion of the Theoretical Solution

A theoretical solution has been developed here that allows the evaluation of the full field response of PSC plates under periodic loads. This solution is based on the Fourier series expansion of the boundary conditions and uses an extended Stroh’s method to construct the solution. The following shows some of the characteristics of this solution corresponding to the constant term and exponential terms.

### 4.1. Influence of the Constant Boundary Condition

There is a part of the solution that comes from the constant terms. This part exhibits physical field properties that are more intuitive and are prominent when the PSC plate is subjected to uniform loads applied along the $x_{3}$ direction. For general elastic and piezoelectric materials, these terms result in physical fields that are constant or linearly vary. However, exponential terms appear in this part of the solution for the PSC plate, which causes the attenuation of physical fields from each surface toward the center. In Equation (29), the expression with factor $k_{0}$ indicates that these exponential terms are produced by the multi-field coupling effect of the PSC plate and that the attenuation will be more intense when the concentrations of the two types of carriers are larger. Moreover, the electric displacement component is the only non-zero quantity in $b_{0}$, which reveals that the electric field delivers the coupling effect. An example of the PSC plate under uniform tensile stress derived from refs. [23,39] is used to verify this conjecture. It should be noted that, in this problem, there are no concentrated charges and there are no currents flowing through the two surfaces of the plate; thus, the boundary conditions for the problem are:

(41) $$\begin{array}{l} t_{3}(-h)=t_{3}(0)=\sigma_{0},\\ D_{3}(-h)=D_{3}(0)=0,\\ J_{3}^{n}(-h)=J_{3}^{n}(0)=0. \end{array}$$

**Figure 7:**
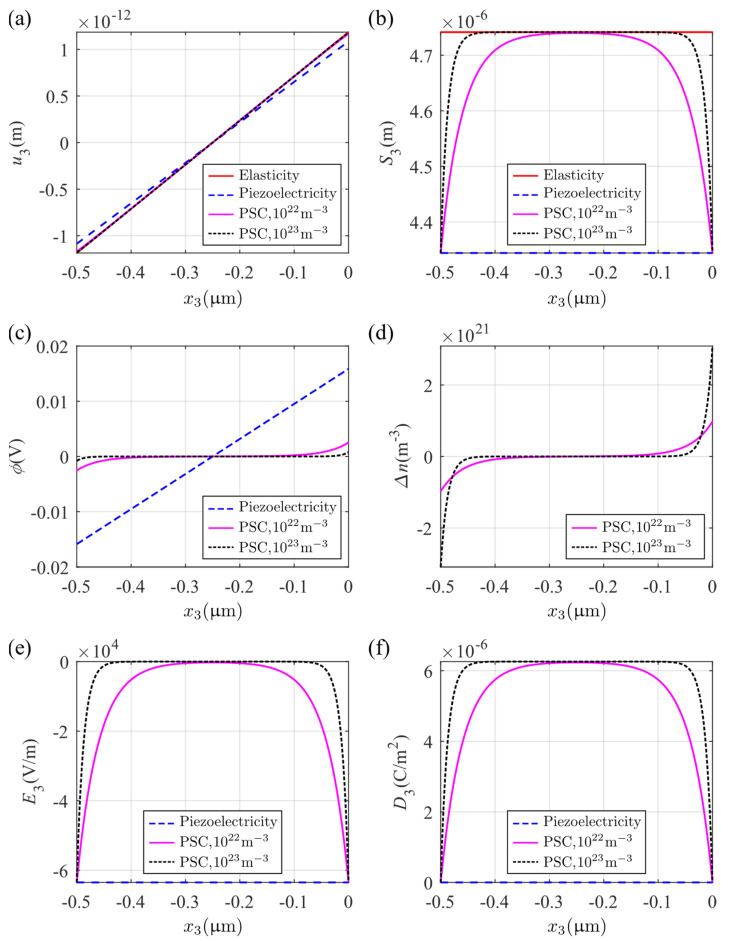
Distributions of physical fields under a uniform tensile stress (electrically isolated): (**a**) mechanical displacement $u_{3}$, (**b**) axial strain $S_{3}$, (**c**) electric potential φ, (**d**) perturbation of electron concentrations Δn, (**e**) electric field $E_{3}$, and (**f**) electric displacement $D_{3}$.

The thickness of the plate in this case is increased to h=0.5 μm in order to clearly show the change in physical fields in the thickness direction; we have used $\sigma_{0}=1\times 10^{6}{\;N/m}^{2}$. The material considered is an n-type PSC with two different initial carrier concentrations, $n_{0}=1\times 10^{22}{\;m}^{-3}$ and $n_{0}=1\times 10^{23}{\;m}^{-3}$. In addition, for the same load, the cases that degenerate to piezoelectric and purely elastic materials are also considered. Figure 7 shows the distribution of the physical fields along the $x_{3}$ axis for different initial concentrations and for the special cases of a purely piezoelectric plate and a purely elastic plate. Figure 7a,b show the mechanical displacement and strain, demonstrating how the PCS provides transitions from the piezoelectric at the boundaries towards the purely elastic area at the center. This transition is more abrupt as $n_{0}$ increases. The transition of the PSC response to the piezoelectric on approaching the boundary is seen also in Figure 7e,f for, respectively, the electric field and electric displacement. As indicated in Figure 7e, the electric field for the PSC develops (becomes non-zero) as one moves to the boundaries while the electric displacement for the PSC is zero at the boundaries and becomes non-zero as one moves to the interior of the bar, indicating that the dominance of the piezoelectricity effect in the PSC attenuates from the surface to the interior, with the transition being more pronounced as $n_{0}$ becomes larger. The electric potential shown in Figure 7c indicates that the electromechanical coupling in this material is weaker than that of general piezoelectric materials and it decays with the increase in $n_{0}$. Figure 7d shows that a larger $n_{0}$ makes the perturbation of electron concentrations more concentrated on the surfaces. Combined with the response in Figure 7e, this indicates that the conductivity of the material is exaggerated while the piezoelectricity is weakened.

### 4.2. The Effect of Reciprocal Length ξ

Other than the part of the solution that comes from the constant term, the balance corresponds to the influence of the remaining exponential terms, which are related to the loading period length. The following analysis is based on solving an extended Stroh’s eigenvalue problem. It can be theoretically shown that all the eigenvalues are non-real; hence, the solution selects a sinusoidal distribution along the $x_{1}$ direction and a decay along the $x_{3}$ direction. Whether the decay is exponential or has an oscillation depends on whether the eigenvalue is complex or pure imaginary. For linear elastic and piezoelectric materials, these eigenvalues only depend on material parameters; whereas, for PSC materials, they also vary with the characteristic reciprocal length ξ. To evaluate this, we study the transversely isotropic PSC material used in the previous section. The zero of the determinant of the coefficients of Equation (18) gives us the reduced characteristic equation for the eigenvalue problem as:

(42) $${\left(p^{2}+1\right)}^{2}\left(c_{44}p^{2}+c_{66}\right)\left(\theta K_{1}\left(p\right)-K_{2}\left(p\right)\right)=0,$$

where $K_{1}(p)$, $K_{2}(p)$, and θ are given as:

$$\begin{array}{c} K_{1}(p)=\left|\begin{array}{cc} c_{44}p^{2}+c_{11} & \left(c_{13}+c_{44}\right)p\\ \left(c_{13}+c_{44}\right)p & c_{33}p^{2}+c_{44} \end{array}\right|,\;K_{2}(p)=\left|\begin{array}{ccc} c_{44}p^{2}+c_{11} & \left(c_{13}+c_{44}\right)p & \left(e_{31}+e_{15}\right)p\\ \left(c_{13}+c_{44}\right)p & c_{33}p^{2}+c_{44} & e_{33}p^{2}+e_{15}\\ \left(e_{31}+e_{15}\right)p & e_{33}p^{2}+e_{15} & -\left(\varepsilon_{33}p^{2}+\varepsilon_{11}\right) \end{array}\right|\\ \theta =\frac{q^{2}\left(p_{0}+n_{0}\right)}{k_{B}T\xi^{2}}. \end{array}$$

**Figure 8:**
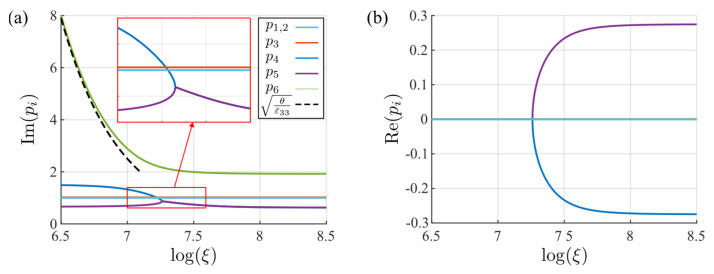
The variation of each eigenvalue with changes to the characteristic reciprocal length parameter ξ: (**a**) imaginary component, (**b**) real component.

The influence of the reciprocal length on the imaginary and real components of the eigenvalues is shown in Figure 8. As the eigenvalues appear in conjugate pairs, in this figure, only the eigenvalues with positive imaginary components are displayed. The figure indicates that three eigenvalues are invariant to changes in the reciprocal parameter. These are the double eigenvalues ($p_{1,2}=i$) that correspond to the drift and diffusion of holes and electrons and the eigenvalue ($p_{3}=i\sqrt{c_{66}/c_{44}}$) that corresponds to the independent displacement component $u_{2}$. The other three eigenvalues, as shown in the last term of Equation (42), depend on the coefficient θ, which is inversely proportional to the square of the characteristic reciprocal length. When θ is large enough, two of these eigenvalues ($p_{4}$, $p_{5}$) tend to the roots of the equation $K_{1}(p)=0$. It can also be shown that under this condition, one eigenvalue ($p_{6}$) is close to $i\sqrt{\theta /\left(\varepsilon_{33}+e_{33}^{2}/c_{33}\right)}$. Conversely, when θ is close to 0, these three eigenvalues tend to be the roots of the equation $K_{2}(p)=0$, which contains real components. In fact, it can be shown that $K_{1}(p)$ and $K_{2}(p)$ are the Stroh’s eigenvalue problems of the corresponding elastic and piezoelectric materials, respectively. Therefore, we can conclude that the piezoelectricity of the PSC material will increase with the characteristic reciprocal length ξ. In other words, the local physical field changes will enhance the electromechanical coupling of the material.

### 4.3. Influence of the Periodic Boundary Conditions

To demonstrate the effect of the period length, we consider a PSC plate with sinusoidal electrical displacement on the upper surface and with a lower surface with zero electric potential. For this demonstration, we set the mechanical traction and current flow equal to zero on both surfaces of the plate. Applying the inverse loads on the negative half-axis segment of the plate is unnecessary in this case. Hence, the boundary conditions on the plate are:

(43) $$\begin{array}{l} t_{3}(x_{1},-h)=t_{3}(x_{1},0)=0,\\ \varphi (x_{1},-h)=0,\\ D_{3}(x_{1},0)=D_{0}\cos\alpha kx_{1},\\ J_{3}^{p}(x_{1},-h)=J_{3}^{p}(x_{1},0)=0,\\ J_{3}^{n}(x_{1},-h)=J_{3}^{n}(x_{1},0)=0. \end{array}$$

**Figure 9:**
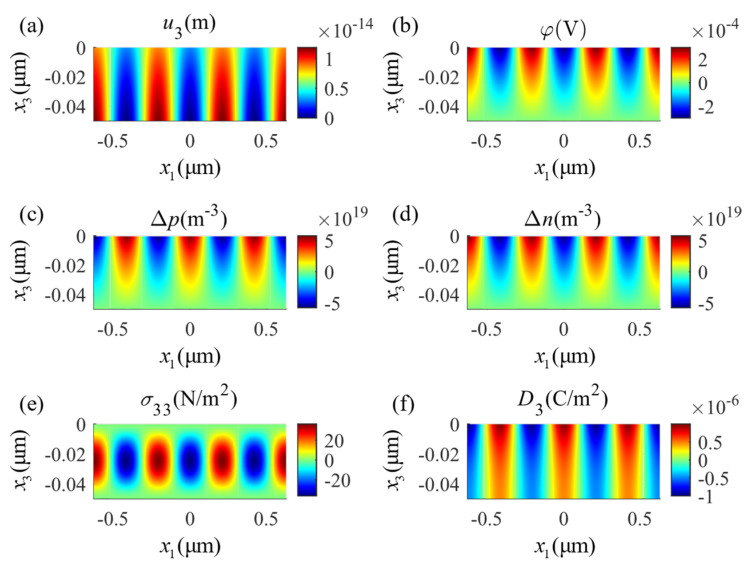
Distributions of physical fields under a sinusoidal electrical field (α=3) applied on the upper surface: (**a**) mechanical displacement $u_{3}$, (**b**) electric potential φ, (**c**) perturbation of hole concentrations Δp, (**d**) perturbation of electron concentrations Δn, (**e**) stress component $\sigma_{33}$, and (**f**) electric displacement $D_{3}$.

The same dimensions for the plate are used as those in the previous example and $D_{0}=1\times 10^{-6}{\;C/m}^{2}$. The parameter α is used to control the period length of the sinusoidal function. In this case, the series solution degenerates to only one term, which exactly corresponds to α. Figure 9 shows the response for α=3 using the general solution given by Equation (29). These indicate that the sinusoidal electrical field on the upper surface causes similar sinusoidal distributions of the electrical parameters, such as those seen in Figure 9b for the electric potential φ and in Figure 9f for the electric displacement component $D_{3}$. These electrical parameters are largest on the upper surface and gradually decrease along the plate thickness to vanish on the lower surface. As noted before, the concentration of electrons in Figure 9d shows the same distribution as the electric potential, while the opposite is true for the concentration of holes indicated in Figure 9c. Due to the electromechanical coupling effect of the structure, the sinusoidal electrical field on the upper surface induces an increasing mechanical displacement $u_{3}$ along the lower surface as shown in Figure 9a. However, the stress component $\sigma_{33}$, shown in Figure 9e, indicates a distribution that fluctuates with the largest fluctuation in the middle of the plate.

**Figure 10:**
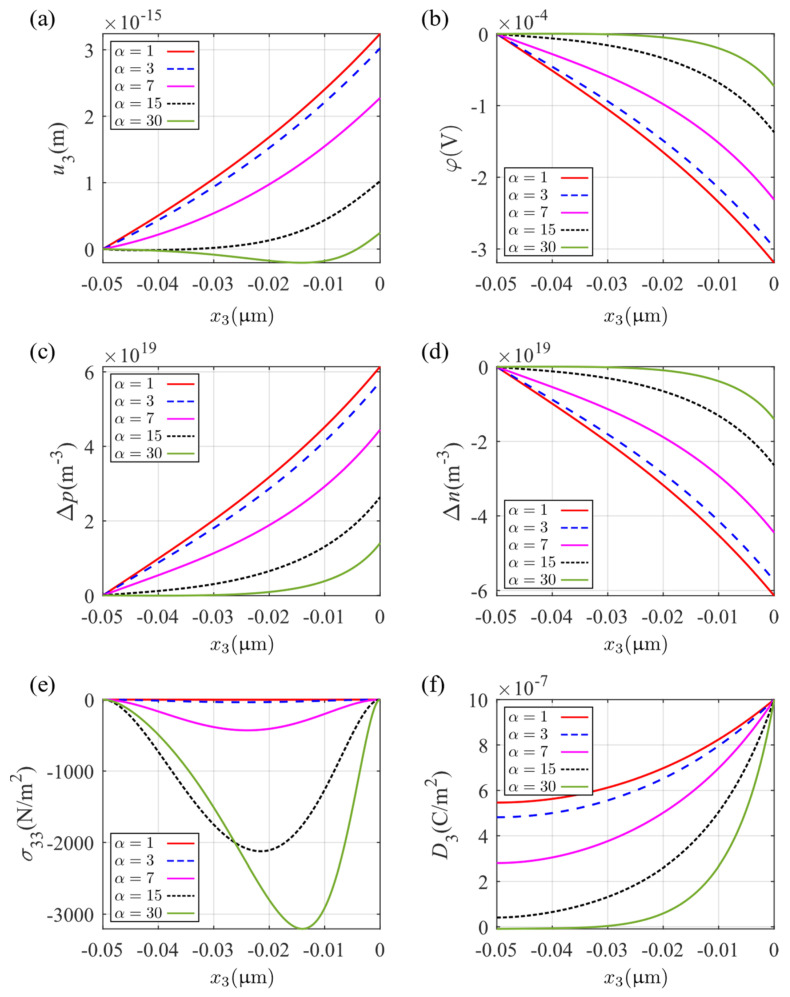
Distribution of physical fields at $x_{1}=0$ along the $x_{3}$ axis: (**a**) mechanical displacement $u_{3}$, (**b**) electric potential φ, (**c**) perturbation of hole concentrations Δp, (**d**) perturbation of electron concentrations Δn, (**e**) stress component $\sigma_{33}$, and (**f**) electric displacement $D_{3}$.

The effect of the size of the fluctuation in the sinusoidal electrical field is demonstrated in Figure 10 by varying α. The figure shows the variation along the thickness variable $x_{3}$ at $x_{1}=0$. On this line, the displacement is shown in Figure 10a, which gradually changes with increasing α from monotonically increasing to oscillating, which verifies the previous discussion on the eigenvalues. With increasing α, stress $\sigma_{33}$ shows higher fluctuation between the free stress boundaries on the two surfaces (Figure 10e), and, with the peak $\sigma_{33}$, they are moving from the middle to the upper surface of the plate. Increasing α induces electric components that have smaller amplitudes on the upper surface. This can be seen, for example, in the electric potential φ (Figure 10b) and the perturbations of electron and hole concentrations Δp and Δn (Figure 10c,d). The electric displacement $D_{3}$ decays faster with increasing α from the set value at the upper surface. These indicate that the effect of the higher-order periodic parts of the electric field on the generalized displacement is gradually weakened; however, the solution also induces the development of larger internal stresses in the plate.

In general, the physical field distributions in PSC plates will always show a trend that indicates non-linear changes from the surfaces toward the interior. This is seen, for example, for the electric potential, which changes sharply near the surface but is flat in the central region while the potential displacement is more likely to increase in the interior. In the solution, as a result of the Fourier series expansion of the boundary conditions, the components with varying eigenvalues have different degrees of attenuation. The extent to which the physical fields interact with each other, which is due to the multi-field coupling effect of the PSC material, typically results in more complicated distributions of the response terms.

## 5. Conclusions

An analytical formulation for calculating the physical field distributions in a thick PSC plate under arbitrary boundary conditions has developed using an extension of Stroh formalism. This extension incorporates the additional fields in the PSC plate. The method, which is based on the Fourier series expansion of the boundary terms, is developed for piezoelectric semiconductor responses and applied to constructing a general solution for a thick PSC plate. The examples of a plate with mechanical four-point bending and a plate with a limit to three-point bending are used to demonstrate the physical field distributions in a thick PSC plate. Results show that the mechanical loads cause complex physical field distributions inside the plate. Among them, the carrier concentrations show the same distribution as the electric potential when there is no outward/inward current. Subsequently, the effects of the initial carrier concentration and reciprocal length on the theoretical solution are discussed; they are related to the physical properties of the material. Uniform tension is studied to evaluate the difference between the PSC, pure elastic, and pure piezoelectric materials. This is conducted in the context of evaluating the effect of changing the initial carrier concentration on the material properties. Results show that a high initial carrier concentration will weaken the piezoelectricity of the material and make the physical field variation more concentrated at the surfaces. The effect of the characteristic reciprocal length on the eigenvalues is studied analytically and the results show that the material exhibits more piezoelectricity as the characteristic reciprocal length increases, causing some of the physical fields to oscillate. The example of applying a variable period sinusoidal electric field on the upper surface further verifies this observation. The examples and discussions indicate that the method is suitable for considering and understanding the effects of complex boundary conditions, particularly when accurate physical field distributions in the plate are required. After building more complicated boundary conditions, more relationships among electric fields, carrier redistributions, and piezoelectricity for PSC may be discovered. The method can also be considered for extension to address dynamic problems by including time items and bias electric field items in a manner similar to that given in refs. [37,38]. In addition, the ability to easily parameterize the loading allows the method to be used for PSC nanodevice design.

## Figures and Tables

**Table 1:** Material coefficients of ZnO.

**Stiffness** $\left(10^{10}\;{N/m}^{2}\right)$				
$c_{11}$	$c_{33}$	$c_{44}$	$c_{66}$	$c_{13}$
20.97	21.09	4.247	4.43	10.51
**Piezoelectric Stress Constants** $\left({C/m}^{2}\right)$			**Dielectric Constants** $\left(10^{-11}\;{F/m}^{2}\right)$	
$e_{15}$	$e_{31}$	$e_{33}$	$\varepsilon_{11}$	$\varepsilon_{33}$
−0.48	−0.573	1.32	7.57	9.03

## Data Availability

The data presented in this study are available on request from the corresponding author.
